# Supplementary material for: Association of time-averaged serum uric acid level with clinicopathological information and long-term outcomes in patients with IgA nephropathy
Source: PeerJ. 2024 Apr 19;12:e17266. doi: 10.7717/peerj.17266 (PMC11034505; doi:10.7717/peerj.17266)
Supplement: Figure S1 [file peerj-12-17266-s001.docx]

**Supplemental Figure 1.** Serial measurements of proteinuria during follow-up in one patient and the definition of time-averaged serum uric acid (TA-SUA) for this patient.

**Supplemental Figure 2.** The clinical data and treatments during follow-up of IgA nephropathy patients in four groups according to quartiles of time-averaged serum uric acid (TA-SUA): (A) TA-SUA; (B) TA-hematuria; (C) TA-serum albumin; (D) the occurrence of proteinuria remission during first 6 months; (E) the use of a RAAS inhibitor; and (F) the use of a glucocorticoid.

**Supplemental Figure 3.** The associations between time-averaged serum uric acid (TA-SUA) level and serum complement features among the male and female patients with IgA nephropathy. TA-SUA correlated with (A) serum C3 (male: *r*=0.113, *P*=0.329; female: *r*=0.142, *P*=0.222); and (B) serum C4 (male: *r*=0.209, *P*=0.070; female: *r*=0.112, *P*=0.341).

**
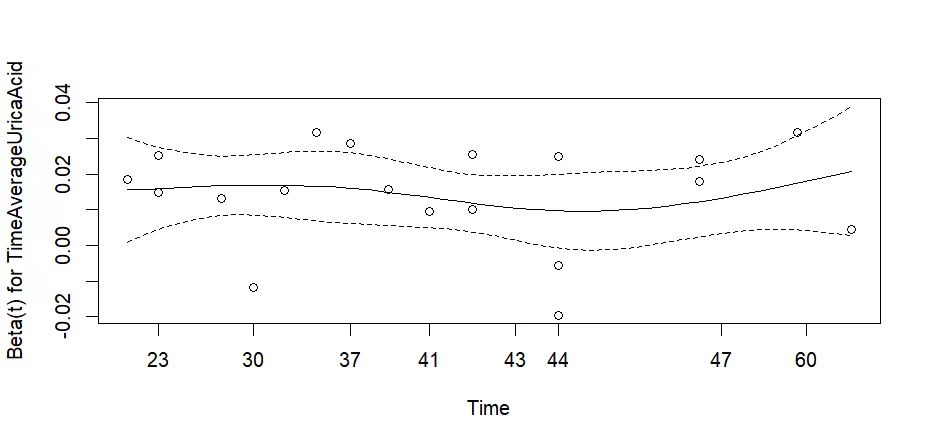
**

**Supplemental Figure 4.** The proportional hazards assumption based on Schoenfeld residuals. *P* >0.05.
